# MYEOV overexpression induced by demethylation of its promoter contributes to pancreatic cancer progression via activation of the folate cycle/c-Myc/mTORC1 pathway

**DOI:** 10.1186/s12885-022-10433-6

**Published:** 2023-01-25

**Authors:** Shoichiro Tange, Tomomi Hirano, Masashi Idogawa, Eishu Hirata, Issei Imoto, Takashi Tokino

**Affiliations:** 1 Department of Medical Genome Sciences, Research Institute for Frontier Medicine, Sapporo Medical University School of Medicine, S1W17, Chuo-ku, Sapporo, 060-8556 Japan; 2 Department of Gastroenterology and Hepatology, Sapporo Medical University School of Medicine, S1W16, Chuo-ku, Sapporo, 060-8543 Japan; 3 Division of Tumor Cell Biology and Bioimaging, Cancer Research Institute, Kanazawa University, Kakuma-machi, Kanazawa, 920-1192 Japan; 4 Aichi Cancer Center Research Institute, 1-1 Kanokoden, Chikusa-ku, Nagoya, 464-8681 Japan

**Keywords:** TCGA, Pancreatic cancer, DNA methylation, C-Myc, mTORC1

## Abstract

**Background:**

While molecular targeted drugs and other therapies are being developed for many tumors, pancreatic cancer is still considered to be the malignant tumor with the worst prognosis. We started this study to identify prognostic genes and therapeutic targets of pancreatic cancer.

**Methods:**

To comprehensively identify prognostic genes in pancreatic cancer, we investigated the correlation between gene expression and cancer-specific prognosis using transcriptome and clinical information datasets from The Cancer Genome Atlas (TCGA). In addition, we examined the effects of the suppression of candidate prognostic genes in pancreatic cancer cell lines.

**Result:**

We found that patients with high expression levels of *MYEOV*, a primate-specific gene with unknown function, had significantly shorter disease-specific survival times than those with low expression levels. Cox proportional hazards analysis revealed that high expression of *MYEOV* was significantly associated with poor survival and was an independent prognostic factor for disease-specific survival in pancreatic cancer patients. Analysis of multiple cancer samples revealed that the *MYEOV* promoter region is methylated in noncancer tissues but is demethylated in tumors, causing *MYEOV* overexpression in tumors. Notably, the knockdown of *MYEOV* suppressed the expression of *MTHFD2* and other folate metabolism-related enzyme genes required for the synthesis of amino acids and nucleic acids and also restored the expression of c-Myc and mTORC1 repressors.

**Conclusion:**

There is a significant correlation between elevated *MYEOV* expression and poor disease-specific survival in pancreatic cancer patients. *MYEOV* enhances the activation of several oncogenic pathways, resulting in the induction of pancreatic cancer cell proliferation. Overall, *MYEOV* acts as an oncogene in pancreatic cancer. Furthermore, *MYEOV* may be a prognostic biomarker and serve as an ‘actionable’ therapeutic target for pancreatic cancers.

**Supplementary Information:**

The online version contains supplementary material available at 10.1186/s12885-022-10433-6.

## Background

With the clinical introduction of novel therapies such as molecular targeted drugs and immune checkpoint inhibitors for many malignancies, the overall mortality rate of cancer patients is decreasing, but pancreatic cancer is still considered one of the malignancies with the poorest prognosis [1]. Pancreatic cancer is also predicted to become one of the major causes of cancer-related death in the near future because it can metastasize to other organs by the time the diagnosis is confirmed and due to the lack of treatment options. In recent years, therapies such as FOLFIRINOX have been applied and have shown good efficacy and survival improvement compared to gemcitabine monotherapy. Immune checkpoint inhibitors have also been approved for use in some pancreatic cancers, but effective molecular targeted therapy has yet to be developed. To identify new molecular markers or candidate therapeutic targets in pancreatic cancer, we searched for genes whose expression was significantly correlated with shorter disease-specific survival in the expression profiles of RNA-Seq datasets of pancreatic cancer samples in The Cancer Genome Atlas (TCGA) because we have enough experience to perform such analysis [2, 3]. Gene screenings using overall survival information have been reported by other groups. Therefore, we newly performed screening using disease-specific survival information because overall survival information considers patients who died from causes other than the disease being studied. Generally, disease-specific survival can characterize outcomes best among various survival metrics. This screening revealed that patients with pancreatic cancer overexpressing *MYEOV*, named the myeloma overexpression gene [4], had significantly shorter disease-specific survival than those with low levels of *MYEOV*. *MYEOV* is known to be overexpressed not only in myeloma but also in breast, oral, esophageal, lung and colorectal cancers [5–9]. In recent years, similar results have been reported in pancreatic cancer as in other malignant tumors [10–12]. The protein encoded by the *MYEOV* gene contains a hydrophobic region and a characteristic C-terminal poly-leucine/isoleucine sequence [4], suggesting that the protein localizes to the cell membrane. However, the biological role of *MYEOV* is poorly understood. This may be due in part to the fact that *MYEOV* is an acquired gene in primates [13], making it difficult to conduct carcinogenesis in mouse models.

In this study, we report that *MYEOV* expression is significantly upregulated at the transcriptional level in pancreatic cancer tissues and that the expression of *MYEOV* is a prognostic indicator that correlates with shorter disease-specific survival independent of lymph node metastasis. Furthermore, knockdown of *MYEOV* induced the downregulation of the c-Myc and mTORC1 pathways, as well as the folic acid metabolism containing their target genes. These data emphasize the potential role of *MYEOV* as a therapeutic target.

## Methods

### Acquisition of information from public databases

Among the transcriptome data of pancreatic cancer specimens, read-count data for RNA-sequencing (RNA-Seq) and DNA methylation analysis were obtained from the TCGA. For the transcriptome data of normal pancreatic tissues, count data were obtained from the Genotype-Tissue Expression (GTEx) database [14], and transcripts per million (TPM) values were calculated using an in-house script. Clinical information, including data on tumor-specific survival in TCGA pancancer datasets, was obtained from cBioPortal.

### Identification of prognostic genes

We used RNA-seq read-count data from pancreatic adenocarcinoma (PAAD) patients for screening. Based on these values, we calculated TPM values and divided the samples into “high expression” and “low expression” groups for each gene using the median value as a cutoff. The presence of a significant difference in disease-specific survival between these two groups was assessed by the log-rank test. Among the genes, those with more events in the high expression group, i.e., death of a patient due to pancreatic cancer, were selected as candidate prognostic markers.

### Cell culture

The pancreatic cancer cell lines AsPC-1 and BxPC-3 (American Type Culture Collection, ATCC) were cultured in RPMI-1640 (FUJIFILM Wako Pure Chemical Co.), MiaPaCa-2 (American Type Culture Collection, ATCC) and Panc-1 (American Type Culture Collection, ATCC) cells were cultured in DMEM (FUJIFILM Wako Pure Chemical Co.), and SUIT-2 (Japanese Collection of Research Bioresources (JCRB), Ibaraki, Japan) cells were cultured in EMEM (FUJIFILM Wako Pure Chemical Co.) containing 10% FBS (Sigma). These cells were authenticated by the STR analysis service of BEX Corporation Ltd.

### 5-aza-2′-deoxycytidine (5-aza-dC) treatment

MiaPaCa-2 cells were cultured with DMEM containing 1, 2 and 5 μmol/L 5-Aza-dC (FUJIFILM Wako Pure Chemical Co.) or DMSO as a control for 7 days. After 7 days of culture, the cells were collected, and total RNA was extracted as described below.

### Small interfering RNA (siRNA) transfection

siRNAs targeting *MYEOV* (Ambion #s25555, #s25556) and control siRNA (Ambion #4390843) were introduced into cells at a final concentration of 10 nM using OptiMEM (Gibco) and Lipofectamine RNAi MAX (Invitrogen). The sequences of the siRNAs are shown in Table S1.

### Evaluation of proliferative capacity

Cell proliferation was assessed at the indicated time after seeding (1 × 10^3^ or 2 × 10^3^ cells/96-well plate) using the RealTime-Glo MT Cell Viability Assay kit (Promega). The luminescence intensities were acquired with a Powerscan H1 microplate reader (DS Pharma Biomedical). The results were expressed as the average absolute absorbance at the indicated time divided by the average relative luminescence unit of each sample incubated for 24 hours after seeding.

### RNA extraction and gene expression analysis

RNA was extracted using TRIzol (Invitrogen) or an RNeasy mini kit (Qiagen) and was further reverse-transcribed with ReverTra Ace qPCR RT Master Mix with gDNA Remover (Toyobo). RNA from normal pancreatic tissue was purchased from Biochain, and cDNA was prepared using the same method as above. Gene expression levels were measured by quantitative PCR (qPCR) using GeneAce qPCR (Nippongene) and QuantStudio3 Real-time PCR system (Thermo) according to the manufacturer’s protocol. Ct values were determined for *TBP* and each gene of interest during the log phase of the cycle. Gene of interest levels were normalized to *TBP* for each sample (dCt = gene of Ct of interest - Ct of *TBP*) and compared to values obtained for known positive controls using the equation Ct, where ddCt = dCt treated - dCt control. The sequences of the primers used for the evaluation are shown in Table S1.

### RNA sequencing (RNA-Seq) analysis

We performed RNA-Seq analysis using the above RNAs. Total RNA was used as a template after confirming that the RNA integrity number (RIN) was approximately 9 using a BioAnalyzer (Agilent). mRNA was extracted using NEBNext Poly(A) mRNA Magnetic Isolation Module (NEB), and NEB NEXT Directional Ultra RNA Library Prep Kit (NEB) was used to prepare the library for sequencing. Stranded paired-end RNA-seq with a read length of 150 bp was performed using NovaSeq (Illumina) according to the manufacturer’s protocol. The acquired sequence reads were aligned with the human genome sequence (hg38) using the 2-PASS-method of STAR (version 2.6.1) [15], and a BAM (Binary Alignment Map) file was generated. The read counts for each gene were quantified using featureCounts (version 2.2.1) [16]. The read counts were further used to detect the differentially expressed genes between control and *MYEOV* knockdown using the DESeq2 package in R [17]. Among the results of DESeq2, genes with *p* < 0.05 were considered differentially expressed genes (Table S4). Comparison of expression levels between samples was done based on transcripts per million (TPM) obtained by TPMcalculator [18].

### Gene set enrichment analysis (GSEA)

GSEA 4.1.0 [19] was used to investigate the biological pathways altered by *MYEOV* knockdown. Gene set files were obtained from the Molecular Signatures Database (mSigDB, https://www.gsea-msigdb.org/gsea/msigdb/). For the analysis, the gene set was permuted 1000 times, and pathways with *p* < 0.05 were considered significantly enriched.

### Western blot analysis

Total cells were lysed with RIPA buffer (Nacalai). The samples were separated by sodium dodecyl sulfate polyacrylamide gel electrophoresis (SDS-PAGE) using 4–15% gradient gels (Bio-Rad) and transferred onto Immobilon-P PVDF membranes (GE). Immunoreactive proteins and phosphoproteins were detected using enhanced chemiluminescence (ECL; Thermo) and the intensity was collected with FUSION imager machine (VILVAR). The antibodies used in this study was listed in Table S2.

## Results

### *MYEOV* expression correlates with a poor prognosis in pancreatic cancer

Using the RNA-Seq data of pancreatic cancers (PAAD) deposited in the TCGA, patients were divided into two groups with a median expression (TPM) value of each gene and examined for correlation with disease-specific survival (Table 1).

**Table 1:** The top 20 genes with a significant correlation between disease-specific survival and expression

Gene ID	Gene name	Number of events in high (***n*** = 86) vs. low (***n*** = 85)		Logrank ***p*** value
ENSG00000172927	MYEOV	48	24	1.79E-06
ENSG00000176485	PLAAT3	47	25	1.92E-05
ENSG00000134333	LDHA	50	22	6.27E-07
ENSG00000265763	ZNF488	47	25	2.49E-05
ENSG00000241749	RPSAP52	48	24	7.32E-06
ENSG00000151491	EPS8	49	23	6.50E-05
ENSG00000105976	MET	49	23	0.0001121
ENSG00000189057	FAM111B	47	25	0.0001129
ENSG00000157456	CCNB2	47	25	3.25E-05
ENSG00000112414	ADGRG6	47	25	1.98E-05
ENSG00000149948	HMGA2	47	25	1.85E-05
ENSG00000276850	AC245041.2	47	25	7.22E-05
ENSG00000065613	SLK	45	27	7.93E-05
ENSG00000170632	ARMC10	49	23	0.0002239
ENSG00000271447	MMP28	45	27	9.95E-05
ENSG00000198901	PRC1	48	24	7.43E-05
ENSG00000092853	CLSPN	49	23	4.04E-05
ENSG00000231991	ANXA2P2	47	25	2.66E-05
ENSG00000075218	GTSE1	50	22	2.12E-05
ENSG00000166889	PATL1	47	25	0.0002436

**Fig. 1:**
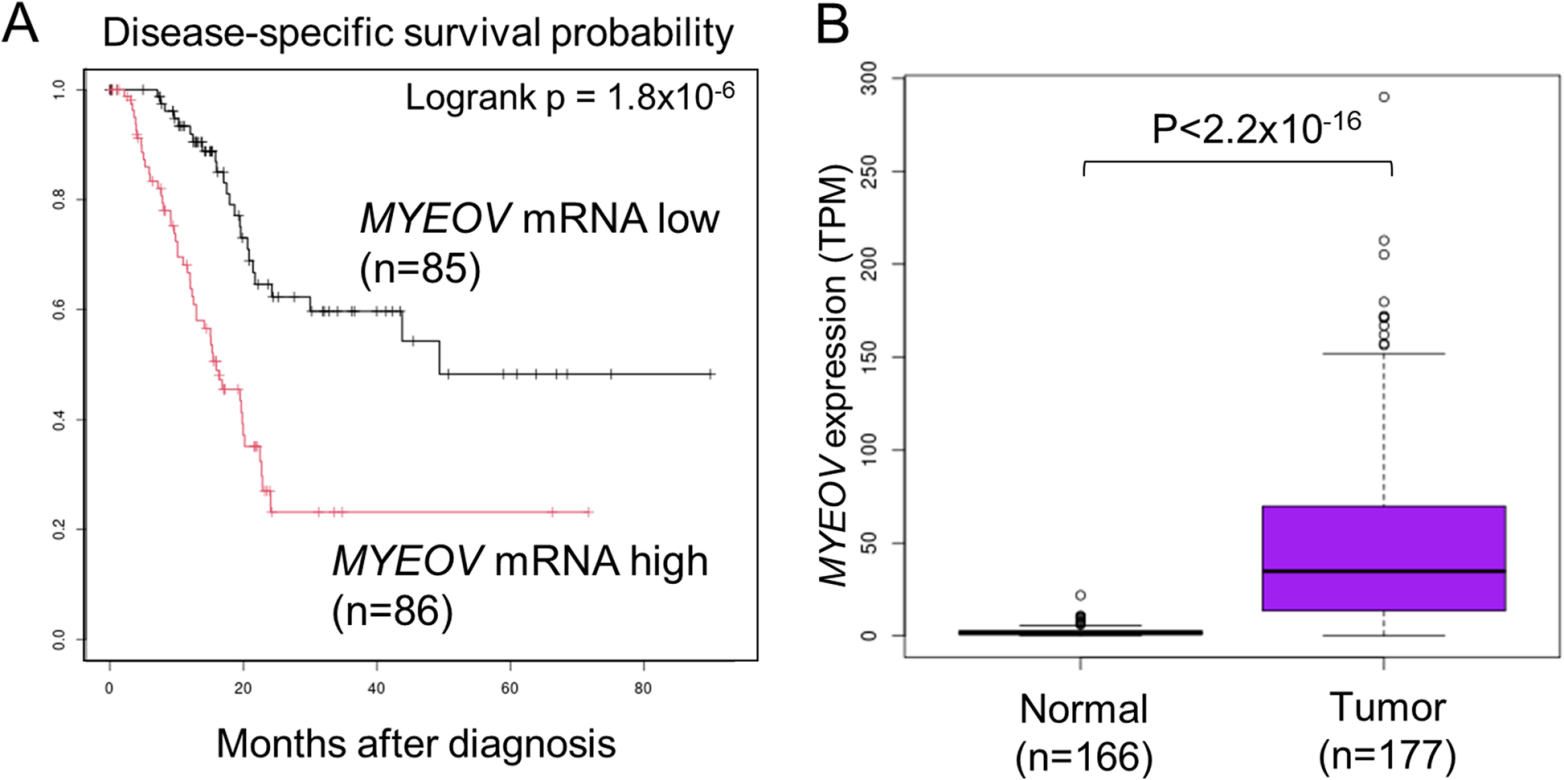
*MYEOV* expression correlates with a poor prognosis and is tumor specific. **A**, Kaplan–Meier plot for the disease-specific survival (DSS) rate of PAAD patients according to *MYEOV* mRNA expression levels. Patients were divided into two groups using the median TPM value of *MYEOV*. The *P* value was calculated using the log-rank test. **B**, Comparison of the expression of *MYEOV* in normal pancreas and pancreatic cancer tissues. Expression data for normal pancreatic tissue were obtained from GTEx. The P value was calculated with the Mann–Whitney U test

The results showed that increased expression of *MYEOV* was significantly correlated with shorter disease-specific survival (Fig. 1A). We performed a similar screen using overall survival information and found that 11 genes out of the top 20 genes, including *MYEOV*, were commonly extracted (Fig. S1A and B). High *MYEOV* expression was also correlated with worse recurrence-free survival and progression-free survival (Fig. S1C and D).

Comparison of gene expression between normal pancreatic tissue in GTEx and TCGA-PAAD RNA-Seq data revealed that the *MYEOV* expression level is extremely low in normal tissues and is specifically upregulated in cancer tissues (Fig. 1 B). We further compared the expression levels of *MYEOV* using TCGA data from other cancers and GTEx normal tissue datasets. Overexpression of *MYEOV* was also observed in bladder, colon, clear renal cell kidney, lung adenocarcinoma, rectum, and stomach cancers (Fig. S3). Cox regression analysis using clinical information such as patient age, tumor size, lymph node metastasis, and sex showed that the expression of the *MYEOV* gene and lymph node metastasis were independent prognostic predictors (Table 2).

**Table 2:** Cox proportional hazard regression analysis of disease-specific survival in patients in the TCGA-PAAD cohort Cox proportional hazard regression analysis of disease-specific survival in patients in the TCGA-PAAD cohort.

	Univariate				Multivariate			
		**95% confidence interval**				**95% confidence interval**		
	**Hazard ratio**	**Lower**	**Upper**	***P*** **value**	**Hazard ratio**	**Lower**	**Upper**	***P*** **value**
MYEOV mRNA expression	2.839	1.726	4.67	**3.95E-05**	3.061	1.846	5.075	**1.45E-05**
Age	1.067	0.652	1.747	0.796	0.999	0.608	1.641	0.995
N stage	2.579	1.384	4.806	**0.003**	1.989	1.049	3.774	**0.035**
T stage	2.611	1.128	6.046	**0.025**	2.252	0.944	5.374	0.067
Sex	1.346	0.844	2.148	0.212	1.428	0.889	2.294	0.141
Bold numbers indicate *p* values that were regarded as statistically significant (p < 0.05).								

### *MYEOV* expression and methylation status in pancreatic cancers

**Fig. 2:**
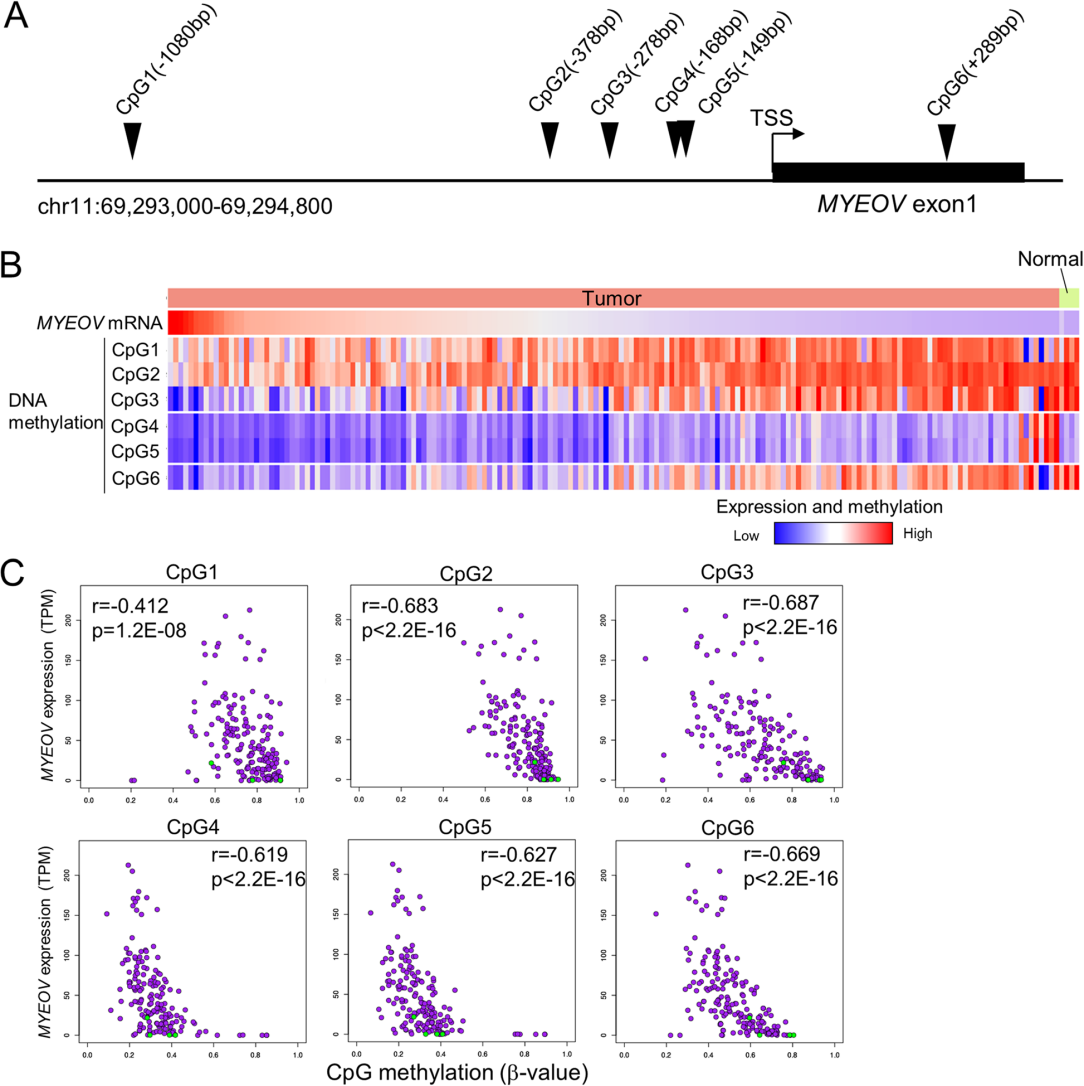
*MYEOV* mRNA expression and DNA methylation status in TCGA-PAAD patients. **A**, Schematic structure of the promoter region of *MYEOV* gene. Arrowheads indicate the location of the CpG region where information on DNA methylation status is recorded in the TCGA. TSS; transcriptional start site. **B**, Heatmap of pancreatic cancer specimens and noncancerous tissues of the TCGA dataset. The expression of *MYEOV* and the methylation status of six CpG sites in the region indicated in A are depicted. Each column represents individual specimens. Specimens are sorted in descending order based on TPM values for *MYEOV*. DNA methylation status is indicated by β-values ranging from 0 to 1 (if cytosine is not methylated at all, the value is 0, and 1 if it is fully methylated). **C**, Correlation between the methylation levels of six CpG regions (x-axis) and the expression levels of *MYEOV* mRNA (y-axis) in PAAD samples from the TCGA dataset. The correlation between methylation and expression levels was calculated by Spearman’s rank correlation coefficient. Purple circle, tumor; green circle, normal

A previous study using esophageal cancer samples reported that the expression of *MYEOV* was inversely correlated with DNA methylation [7]. Therefore, we compared *MYEOV* expression and methylation using DNA methylation information of PAAD samples from the TCGA. According to the analysis of the methylation status of CpG in six probes corresponding to the region from 1080 bp upstream of the transcription start site (TSS) to the first exon (Fig. 2 A), the DNA methylation levels of CpG3 (probe ID: cg01638792) corresponding to 278 bp upstream of TSS and CpG6 (probe ID: cg22779330) in the first exon had a significant inverse correlation with the mRNA expression level of *MYEOV* (Fig. 2 B, C). These locations were also methylated in noncancer pancreatic tissue (Fig. 2 B), suggesting that the loci undergo demethylation during carcinogenesis. In other cancer samples in which *MYEOV* expression was elevated compared to normal tissue, the methylation levels of CpG3 were inversely correlated with mRNA expression. This analysis also showed that CpG3 is highly methylated in normal bladder, colon, kidney, and lung tissues (Fig. S3 and Table S3).

### *MYEOV* expression and methylation status in pancreatic cancer cell lines

**Fig. 3:**
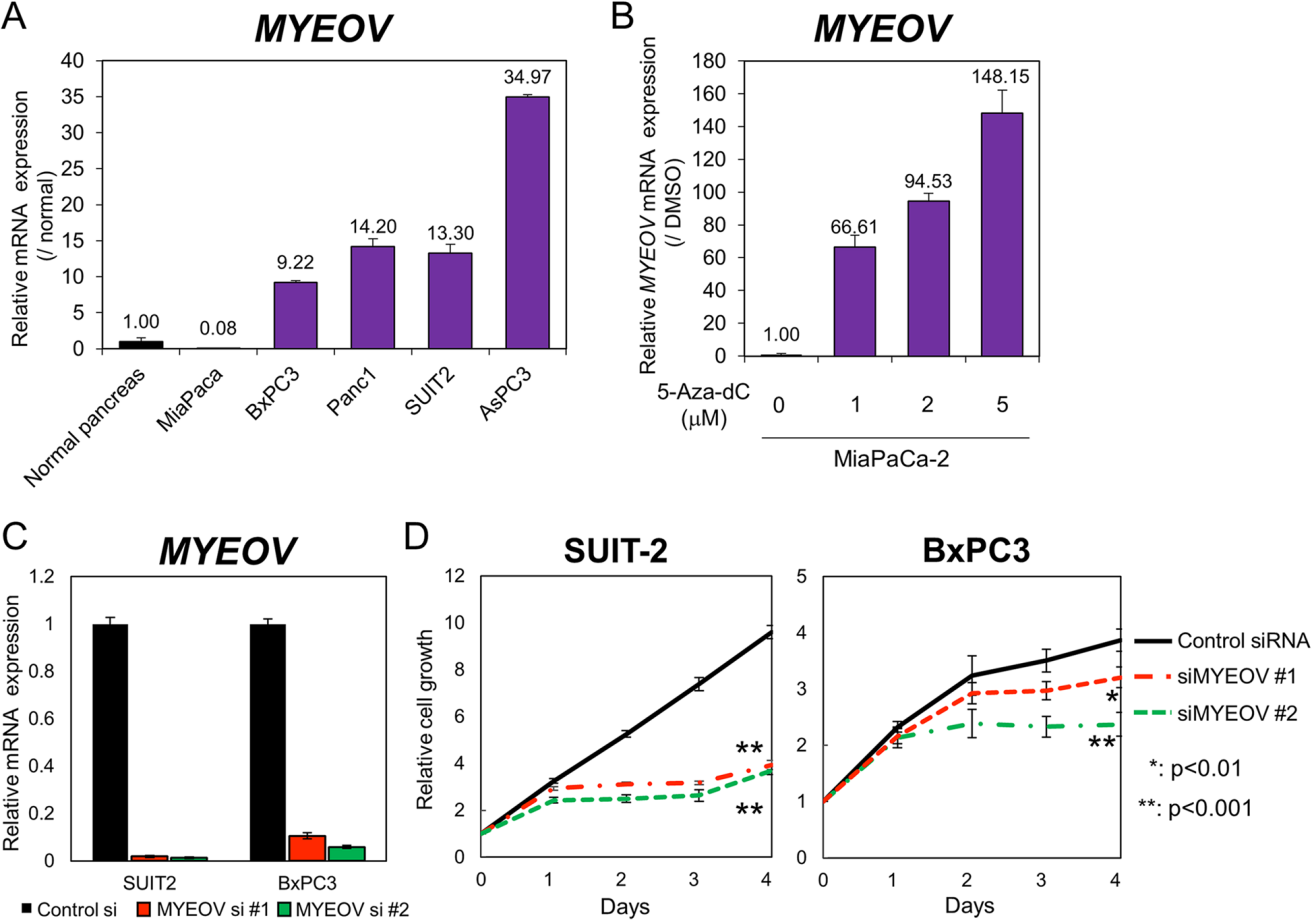
*MYEOV* overexpression in pancreatic cancer cell lines. **A**, cDNA was synthesized from RNA derived from commercially available normal pancreatic tissues or RNA extracted from the five pancreatic cancer cell lines shown in the figure, and the levels of *MYEOV* mRNA were quantified by qPCR. The *MYEOV* mRNA level in normal pancreatic tissues normalized to TBP mRNA was set to 1, and the relative *MYEOV* expression levels in pancreatic cancer cell lines are shown. **B**, Changes in *MYEOV* mRNA expression in the MiaPaCa-2 cell line treated with 5-Aza-dC. Cells were treated with 5-Aza-dC or DMSO for 7 days, and mRNA levels were quantified by qPCR. The amount of *MYEOV* mRNA in each sample was normalized to the amount of *GAPDH* mRNA, and the relative *MYEOV* expression levels are shown in the graph with the DMSO-treated sample as 1. **C**, BxPC3 and SUIT-2 cells were transfected with 10 nM control or *MYEOV*-targeting siRNAs, and the knockdown efficiency was measured by qPCR using *MYEOV*-specific primers. *MYEOV* expression levels are shown in the graph with the nonspecific control siRNA-treated sample as 1. Black bars indicate cells transfected with control siRNA, and red and green bars represent cells transfected with MYEOV-specific siRNA. **D**, Cellular proliferation upon knockdown of *MYEOV* was measured by a real-time GLO growth assay at the indicated time points. The results are expressed as the fold change (mean ± SD, *n* = 4) compared to the respective values of control cells (day 0)

We also examined the expression level of *MYEOV* in pancreatic cancer cell lines by qPCR and found that it was higher in several pancreatic cancer cell lines than in normal pancreatic tissues (Fig. 3 A). In MiaPaCa-2 cells, treatment with 5-Aza-dC, a DNA methyltransferase inhibitor, restored the mRNA expression of *MYEOV* (Fig. 3 B). This result indicates that the expression of *MYEOV* is suppressed by DNA methylation not only in normal pancreatic tissues but also in some pancreatic cancer cells.

### The effects of *MYEOV* knockdown on cellular function in pancreatic cancer cell lines

**Fig. 4:**
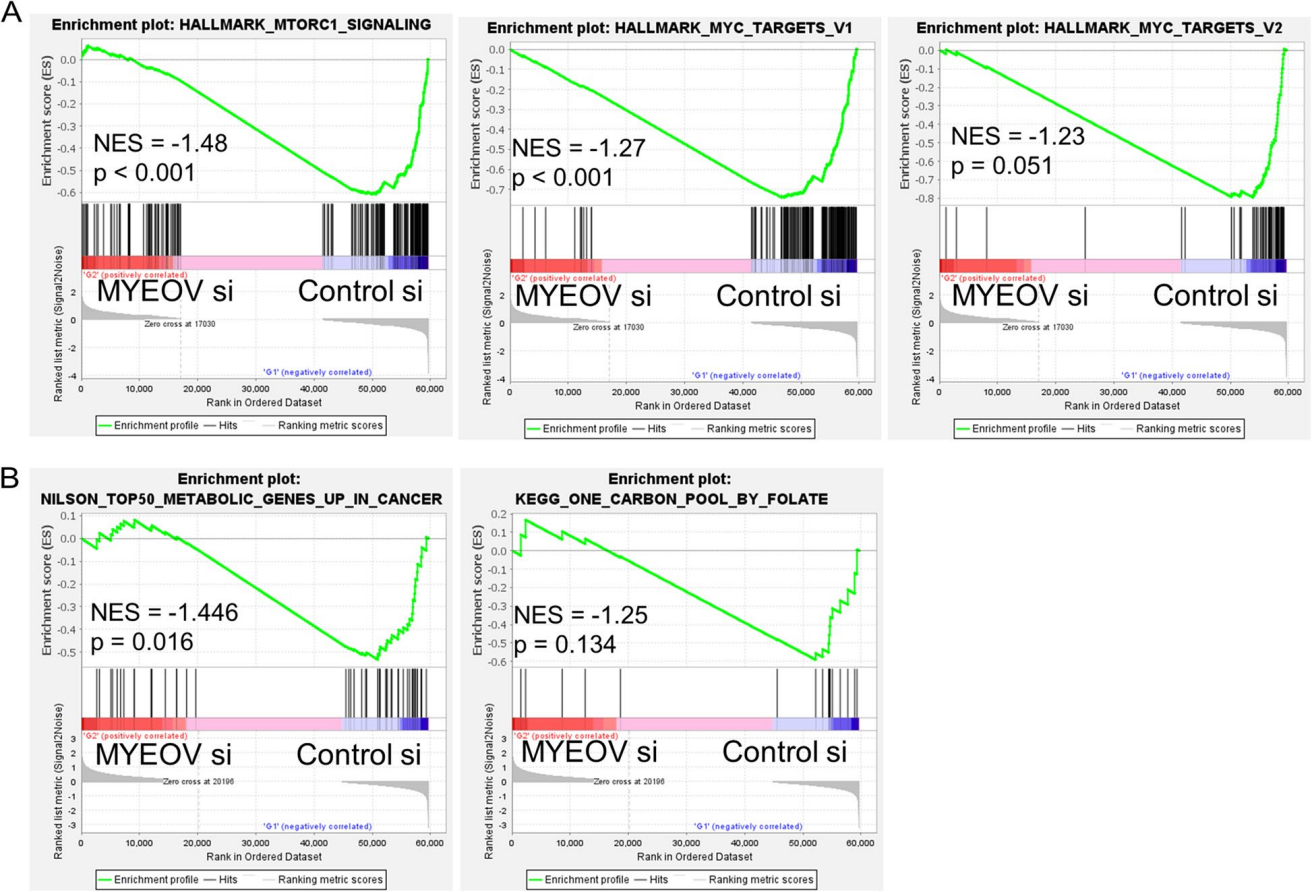
GSEA of *MYEOV* knockdown cells. **A**, The three hallmark gene sets significantly enriched in the control according to GSEA. NES; normalized enrichment score. **B**, The results of GSEA using a gene set of the top 50 metabolism-related genes that are upregulated in cancer (left panel) and genes related to folate metabolic pathways (right panel)

To confirm the roles of *MYEOV* in pancreatic cancer cells, we knocked down *MYEOV* using siRNAs (Fig. 3 C). Knockdown of *MYEOV* decreased the proliferation of several pancreatic cancer cell lines (Fig. 3 D). To verify the molecular mechanism for this change, we performed RNA-Seq using RNA extracted from SUIT-2 cells transfected with siRNAs targeting *MYEOV* or control siRNAs and obtained the gene expression profile as read count data. The read count information was further subjected to GSEA. The analysis showed that the c-Myc target genes and mTORC1 target genes were downregulated by *MYEOV* knockdown (Fig. 4 A and Fig. S4B). To verify the consistency of our results, we further analyzed another RNA-Seq dataset performed by Shen et al. [12] on Panc-1 cells in the same manner as our dataset for the SUIT-2 cell line and performed GSEA using read count values. Similar to our results, the expression of c-MYC target genes and E2F target gene clusters were downregulated by the knockdown of *MYEOV* (Fig. S4B). Of note, the expression level of *MYC* was also significantly reduced by the knockdown of *MYEOV* (Table S4, S5). Based on these results, we analyzed the expression of several c-Myc target genes, and confirmed that the expression of SUIT-2, BxPC-3 and Panc-1 target gene groups tended to be reduced by *MYEOV* knockdown (Fig.S5). We also examined changes in mTORC1 activity by Western blot for the phosphorylation of its substrate, p70 ribosomal protein S6 kinase (p70S6K) [20]. In all three cell types used in the experiment, phosphorylation levels of p70S6K were reduced by the knockdown of the *MYEOV* (Fig.S6).

### The relationship between *MYEOV* and folate metabolism

**Fig. 5:**
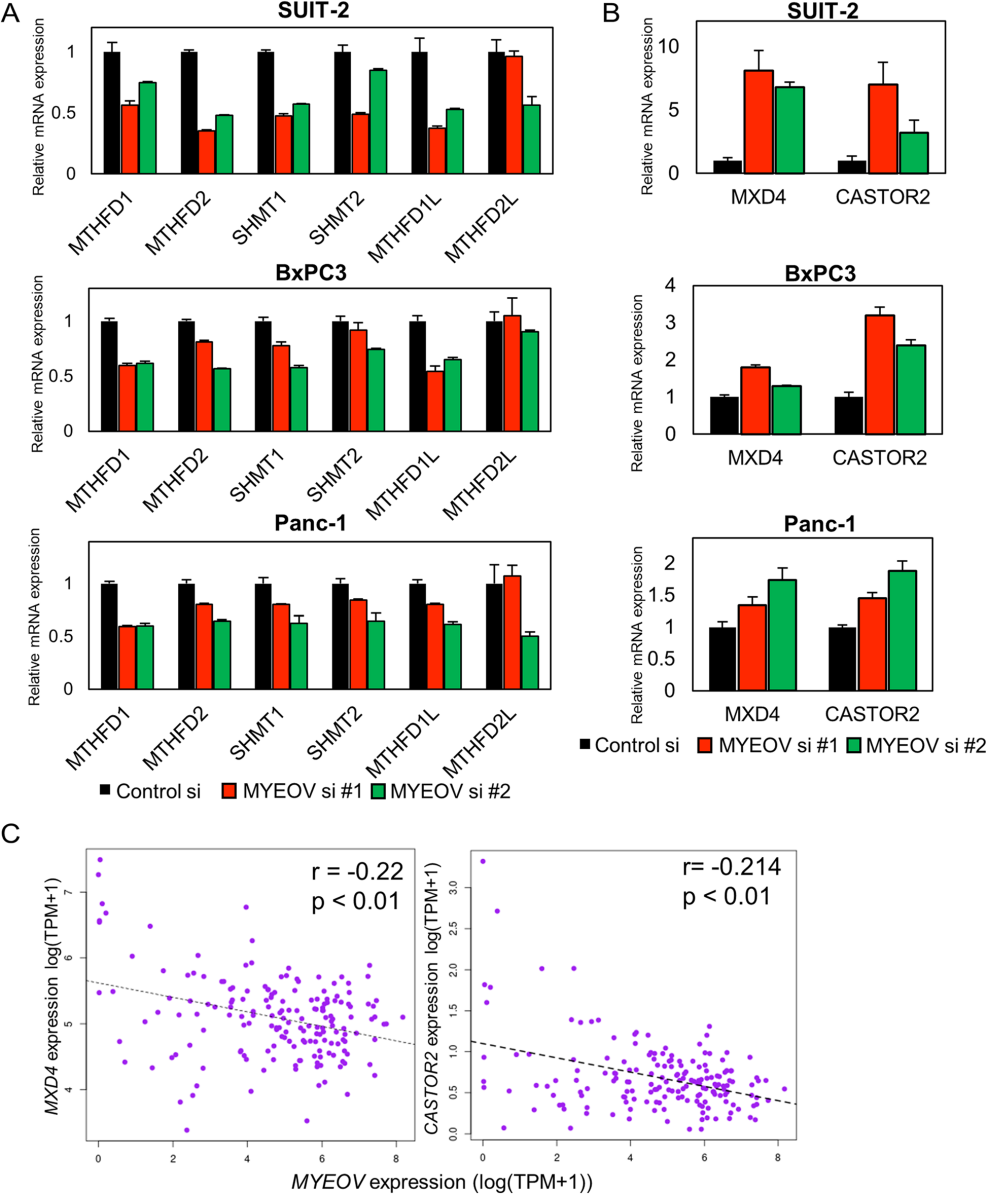
The expression status of genes in the folate cycle upon *MYEOV* knockdown. **A**, The expression levels of genes associated with one-carbon metabolism, *MTHFD1*, *MTHFD2*, *SHMT1*, *SHMT2*, *MTHFD1L*, and *MTHFD2L*, were measured by qPCR in the indicated cell lines. **B**, The expression levels of *CASTOR2* and *MXD4* mRNAs were measured by qPCR. In A and B, black bars indicate cells transfected with control siRNA, and red and green bars represent cells transfected with *MYEOV*-targeting siRNA; *TBP* was used to normalize the expression levels. **C**, Correlations between *MYEOV* and *MXD4* (left) or *CASTOR2* (right) mRNA expression levels in the TCGA-PAAD dataset. Expression levels are shown in terms of log-transformed TPM values, and correlation coefficients and *p* values were calculated using Spearman’s rank correlation coefficient analysis. The dotted lines indicate the correlation regression line

In tumors, the activation of c-Myc and mTORC1 modifies cellular metabolic pathways contributing to tumor growth and survival [21, 22]. In addition, our GSEA results showed that knockdown of *MYEOV* downregulated the expression of metabolism-related genes that were upregulated in tumors (Fig. 4 B, left). While glycolytic genes have already been reported to be downregulated by *MYEOV* knockdown by Tang et al. [10], our result implies that the other metabolic genes are also affected by *MYEOV*. The one-carbon metabolic pathway consists of the folate and methionine metabolic pathways. A group of genes comprising the mitochondrial folate metabolic pathway are usually very low or absent in normal adult tissues but have been reported to be highly expressed in cancer. *MTHFD2*, which comprises this pathway and has two enzymatic activities, dehydrogenase and cyclohydrolase, is reported to be highly expressed at the protein level in a variety of human tumors, including pancreatic cancer, and is negatively correlated with survival [23, 24]. Since *MTHFD2* and other folate metabolism-related genes have been reported to be transcriptional target genes of mTORC1 [25] and c-Myc, we confirmed whether the knockdown of *MYEOV* could reduce the expression of these genes. As expected, GSEA revealed that the expression of several genes in these pathways was reduced by knockdown of *MYEOV* (Fig. 4 B, right). RNA-Seq results showed that *MTHFD1*, *MTHFD2*, and *MTHFD1L*, genes involved in the folate cycle, were significantly reduced by *MYEOV* knockdown (Table S4). qPCR also showed similar results in other pancreatic cancer cell lines, BxPC-3 and Panc-1 (Fig. 5 A).

Interestingly, our RNA-Seq results showed that the expression levels of *CASTOR2* and *MXD4* were upregulated by *MYEOV* knockdown. The protein encoded by *CASTOR2* forms a heterodimer with CASTOR1 protein and suppresses mTORC1 activity by inhibiting GATOR2 function [26]. Moreover, the Mad4 protein encoded by the *MXD4* gene is one of the elements that forms a ternary complex with c-Myc and represses its transcriptional activity by interacting with Max [27], and Mad4 was markedly increased by the repression of *MYEOV* (Fig. 5 B). A negative correlation between *MYEOV* and both *CASTOR2* and *MXD4* was also observed in the RNA-Seq data of the TCGA-PAAD cohort (Fig. 5 C). These results suggest that *MYEOV* contributes to the enhancement of mTORC1 and c-Myc function in pancreatic cancer.

## Discussion

In this report, we show that the expression of *MYEOV* is upregulated in pancreatic cancer and that upregulation of *MYEOV* is significantly correlated with shorter disease-specific survival. Recently, two groups reported that pancreatic cancer patients with higher *MYEOV* expression showed shorter overall survival [10, 11]. In particular, 20 candidate genes for prognostic indicators have been listed by Tang et al. Our study suggests that the expression level of *MYEOV* may be useful as a prognostic marker for survival. However, the candidate prognostic factors shown in Table 2, except for *MYEOV,* did not overlap with the marker candidates shown by Tang et al. This discrepancy is due to the difference in input data. We assessed disease-specific survival instead of overall survival, which includes death events due to factors other than tumors. Therefore, our gene list may indicate more appropriate candidate prognostic factors in pancreatic cancers. The qPCR results showed that the expression of *MYEOV* was upregulated in some pancreatic cancer cell lines compared to normal pancreatic cells (Fig. 3 A). On the other hand, in MiaPaCa-2 cells, *MYEOV* was silenced by methylation of the CpG sites in the promoter region (Fig. 3 B). These results suggest that the decrease in methylation level and the increase in *MYEOV* expression do not occur in all pancreatic cancers. As shown in Fig. 2, this is consistent with the results of the analysis showing that *MYEOV* promoter methylation is maintained in some pancreatic cancer specimens at levels comparable to those in normal pancreatic tissues. The correlation between *MYEOV* expression and DNA demethylation of transcriptional regulatory regions was also confirmed in other tumors (Fig. S3).

GSEA showed that many genes whose expression was altered by knockdown of *MYEOV* were targets of the c-Myc or mTORC1 complex (Fig. 4 A). Fang et al. showed that the expression of genes with c-Myc-binding motifs in their transcriptional regulatory regions was reduced upon knockdown of *MYEOV* using the non-small-cell lung cancer cell line A549 [8]. Our results confirmed similar results in pancreatic cancer. Recently, Tang et al. also reported the relationship between *MYEOV* expression and the c-Myc/mTORC1 signaling pathway [10]. These results suggest that high expression of *MYEOV* in pancreatic cancer tissues is accompanied by increased activity of c-Myc and mTORC1. In both datasets of our and previous study, [12] GSEA analysis showed that the significant enrichment of c-Myc target genes and other genes was commonly observed. However, the mTORC signaling pathway did not show statistically significant reduction in the previous dataset. This may be due to the difference of cell lines used in the experiments and the different knockdown efficiencies in the two data sets, as shown in Fig. S4A. The log-transformed fold change of *MYEOV* expression after its knockdown was − 2.51 in our SUIT-2 RNA-Seq dataset, whereas − 0.86 in the Panc-1 dataset of previous study (Table S4 and Table S5). The low efficiency of *MYEOV* knockdown may be the reason why the Panc-1 RNA-Seq dataset did not show reduced expression of genes in the folate metabolic pathway. Actually, our experiments using Panc-1 cells with high efficacy of *MYEOV* knockdown show the significant reduction of these genes (Fig. 5).

Several metabolic pathways are altered in tumors, which contribute to survival and growth. The relationship between the glycolytic pathway and *MYEOV* has already been reported by other groups [10], but we found that the expression of a gene cluster in one-carbon metabolic pathways was reduced by *MYEOV* knockdown. Folate and methionine are two pathways by which cells produce one-carbon units, which are used for nucleic acid synthesis and methylation reactions. The enzymes involved in the folate cycle are distributed in the mitochondria and cytoplasm. Of these, the *MTHFD2* gene is known to be expressed during embryonic development and is rarely expressed in adult tissues [28]. *MTHFD2* is overexpressed in some tumors and has been shown to contribute to the development of cancer stem cells and is considered a poor prognostic factor in several tumors, including pancreatic cancer [24, 29]. Strikingly, the expression of *MTHFD2* is induced by both c-Myc and mTORC1. Since our analysis suggests that knockdown of *MYEOV* decreases the activity of c-Myc and mTORC1, it is likely that these events lead to a decrease in the expression of several folate metabolism-related genes, including *MTHFD2*. The expression of *MYEOV* is positively correlated with that of a group of genes that comprise the folate pathway (Table S6). Based on these results, the expression of folate metabolism-related genes may be suppressed by *MYEOV* knockdown in other tumors. Our data suggest that the suppression of *MYEOV* expression is a new factor leading to a poor prognosis in pancreatic cancer patients through activation of c-Myc and mTORC1 and metabolic remapping, such as enhancement of the one-carbon metabolic pathway.

Notably, our RNA-Seq results showed that the expression of *MXD4* and *CASTOR2* was markedly upregulated by *MYEOV* knockdown (Fig. 5 B and Table S4). The Mad4 protein represses the transcriptional activity of c-Myc by forming a protein complex with Max [27]. Therefore, repression of *MXD4* expression by *MYEOV* may facilitate the malignant transformation of pancreatic cancer through activation of c-Myc and other proteins. CASTOR2 protein forms a heterodimeric complex with CASTOR1 and binds to GATOR2, which induces mTORC1 activation by inhibiting GATOR1 [26]. Our study showed that knockdown of *MYEOV* increased *CASTOR2* expression in several cell lines, suggesting that *MYEOV* contributes to mTORC1 activation by regulating *CASTOR2* expression. In TCGA-PAAD, the expression levels of *MXD4* and *CASTOR2* were inversely correlated with the expression level of *MYEOV* (Table S6). An inverse correlation between *MYEOV* and *MXD4* expression levels was also observed in lung adenocarcinomas. In gastric cancer, both *CASTOR2* and *MXD4* expression levels were inversely correlated with *MYEOV* expression levels (Table S6). In TCGA dataset of pancreatic cancers, the absolute value of correlation coefficients between *MYEOV* and *MXD* or *CASTOR4* expression are around − 0.2. Although the value is not necessarily high, we consider that this correlation supports our results because the value is above the 80th percentile of all measured correlation coefficients among all genes and is above the 90th percentile of measured correlation coefficients among inversely correlated genes (Fig. S7).

However, *MYEOV* knockdown in Panc-1 RNA-seq dataset from GEO database did not show the significant upregulation of *MXD4*. This result may also be due to the low efficacy of *MYEOV* knockdown (Fig. S4A), or may suggest that the upregulation of *MXD4* is not essential for the downregulation of *MYC* target genes induced by the downregulation of *MYEOV*.

Although some groups report that *MYEOV* contributes to the progression of not only pancreatic cancer but also several other tumors, the detailed functions of the protein encoded by this gene are limited, except for one report stating that it binds to HES1 and promotes the transcriptional activation of SOX9 [11]. This may be due in part to the fact that the MYEOV protein has no known functional domain. A comparative study of multiple animal genomes reported that the *MYEOV* gene is unique among primates only in Catarrhini. The report also notes that *MYEOV* has a very unusual feature as a cancer-related gene: the amino acid sequence significantly differs between humans and apes [13]. Analysis of *MYEOV* expression levels and promoter methylation status in several cancers in the TCGA showed that the promoter region was hypermethylated in normal tissues, while some tumors showed hypomethylation and overexpression. This is a comparatively rare event in cancers because most tumor suppressor genes are generally inactivated by DNA methylation, and this mechanisms is similar to the regulation of long interspersed element-1 (LINE-1), which shows that DNA demethylation and increased expression levels correlate with a poor prognosis in cancer [30]. According to lung cancer cell line experiments, Fang et al. proposed that *MYEOV* functions as a competing endogenous RNA (ceRNA [31]) that inhibits the binding of miRNAs to their targets, thereby contributing to the invasive and metastatic potential of tumors [8].

**Fig. 6:**
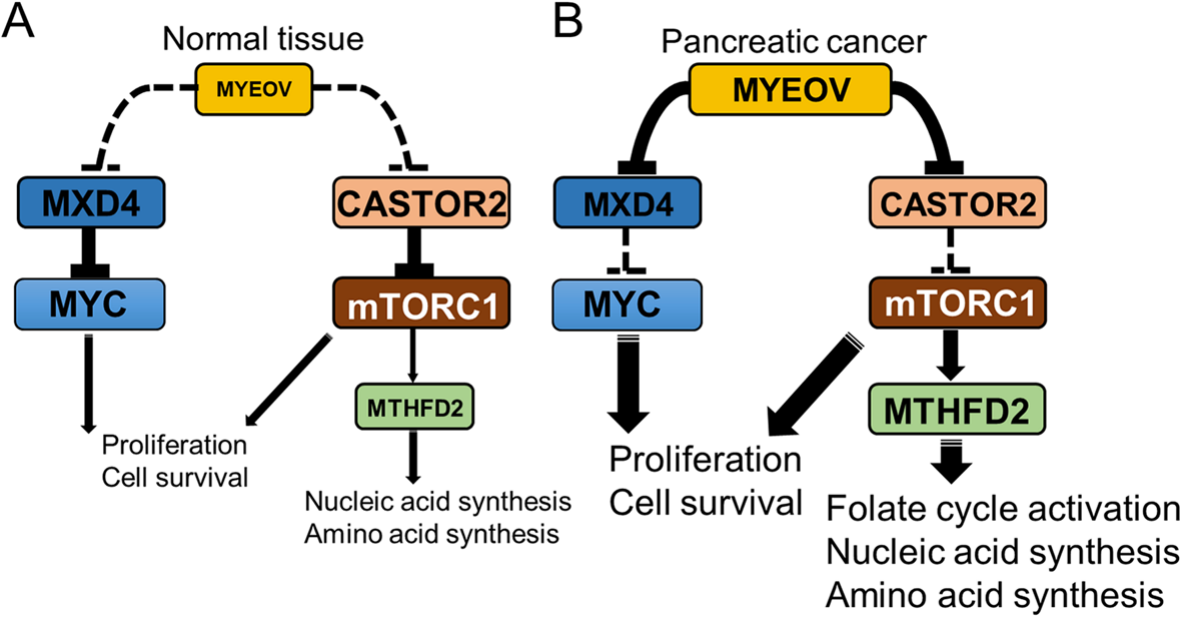
Schematic of the roles of MYEOV in pancreatic cancer. **A**, In normal cells, *MYEOV* expression is low, and *MXD4* and *CASTOR2* appropriately suppress c-Myc and mTORC1. **B**, In tumors, *MYEOV* expression is upregulated and promotes the downregulation of *MXD4* and *CASTOR2*, which induces the activation of c-Myc and mTORC1 and contributes to tumor progression

The outline of our research is shown in Fig. 6. In normal cells, *MYEOV* expression is low. Thus, *MXD4* and *CASTOR2* appropriately suppress c-Myc and mTORC1. Conversely, in pancreatic cancer cells, *MYEOV* expression is upregulated and promotes the downregulation of *MXD4* and *CASTOR2*, resulting in the activation of c-Myc and mTORC1 and tumor progression.

## Conclusions

We sought prognostic markers to identify groups of pancreatic cancer patients with an unfavorable prognosis. In conclusion, we showed that *MYEOV* suppression is a new mechanism that contributes to a poor prognosis in pancreatic cancer patients through the activation of the folate cycle and the c-Myc and mTORC1 pathways (Fig. 6). These studies suggest that *MYEOV* could serve as an ‘actionable’ therapeutic target in several human cancers, including pancreatic cancer. Further research is needed to understand how the MYEOV protein as well as the *MYEOV* transcript itself contribute to tumor progression.

## Supplementary Information

**Additional file 1.**

**Additional file 2.**

**Additional file 3.**

**Additional file 4.**

**Additional file 5.**

**Additional file 6.**

**Additional file 7.**

**Additional file 8.**

**Additional file 9.**

**Additional file 10.**

**Additional file 11.**

**Additional file 12.**

**Additional file 13.**

**Additional file 14.**

**Additional file 15.**

## Data Availability

Gene expression data of the TCGA-PAAD used in our study are available in the Genomics Data Commons repository (https://portal.gdc.cancer.gov/projects/TCGA-PAAD). Gene expression data of the normal pancreas used in our study are available in the GTEx Analysis V7 (dbGaP Accession phs000424.v7.p2, https://storage.googleapis.com/gtex_analysis_v7/rna_seq_data/GTEx_Analysis_2016-01-15_v7_RSEMv1.2.22_transcript_expected_count.txt.gz). The clinical information of TCGA-PAAD patients are available in cBioPortal (https://www.cbioportal.org/study/summary?id=paad_tcga_pan_can_atlas_2018). The RNA-seq dataset performed by Shen et al. [12] using Panc-1 cell line was obtained from the Gene Expression Omnibus (GEO, https://www.ncbi.nlm.nih.gov/gds) under the accession number GSE143827.

## Abbreviations

TCGA: The Cancer Genome Atlas
GTEx: Genotype-Tissue Expression
PAAD: Pancreatic adenocarcinoma
OS: Overall survival
DSS: Disease-specific survival
DFS: Disease-free survival
PFS: Progression-free survival
MYEOV: myeloma overexpression
TPM: Transcripts Per Million
TSS: Transcription start site
GSEA: Gene Set Enrichment Analysis
BLCA: bladder carcinoma
COAD: colon adenocarcinoma
KIRC: renal clear cell carcinoma
LUAD: lung adenocarcinoma
READ: rectum adenocarcinoma
STAD: stomach adenocarcinoma
NES: normalized enrichment score
MTHFD: methylenetetrahydrofolate dehydrogenase
SHMT: serine hydroxymethyltransferase
LINE: long interspersed element
