# Supplementary material for: MYEOV overexpression induced by demethylation of its promoter contributes to pancreatic cancer progression via activation of the folate cycle/c-Myc/mTORC1 pathway
Source: BMC Cancer. 2023 Jan 25;23:85. doi: 10.1186/s12885-022-10433-6 (PMC9875418; doi:10.1186/s12885-022-10433-6)
Supplement: Supplementary file 3 — Additional file 3. [file 12885_2022_10433_MOESM3_ESM.pdf]

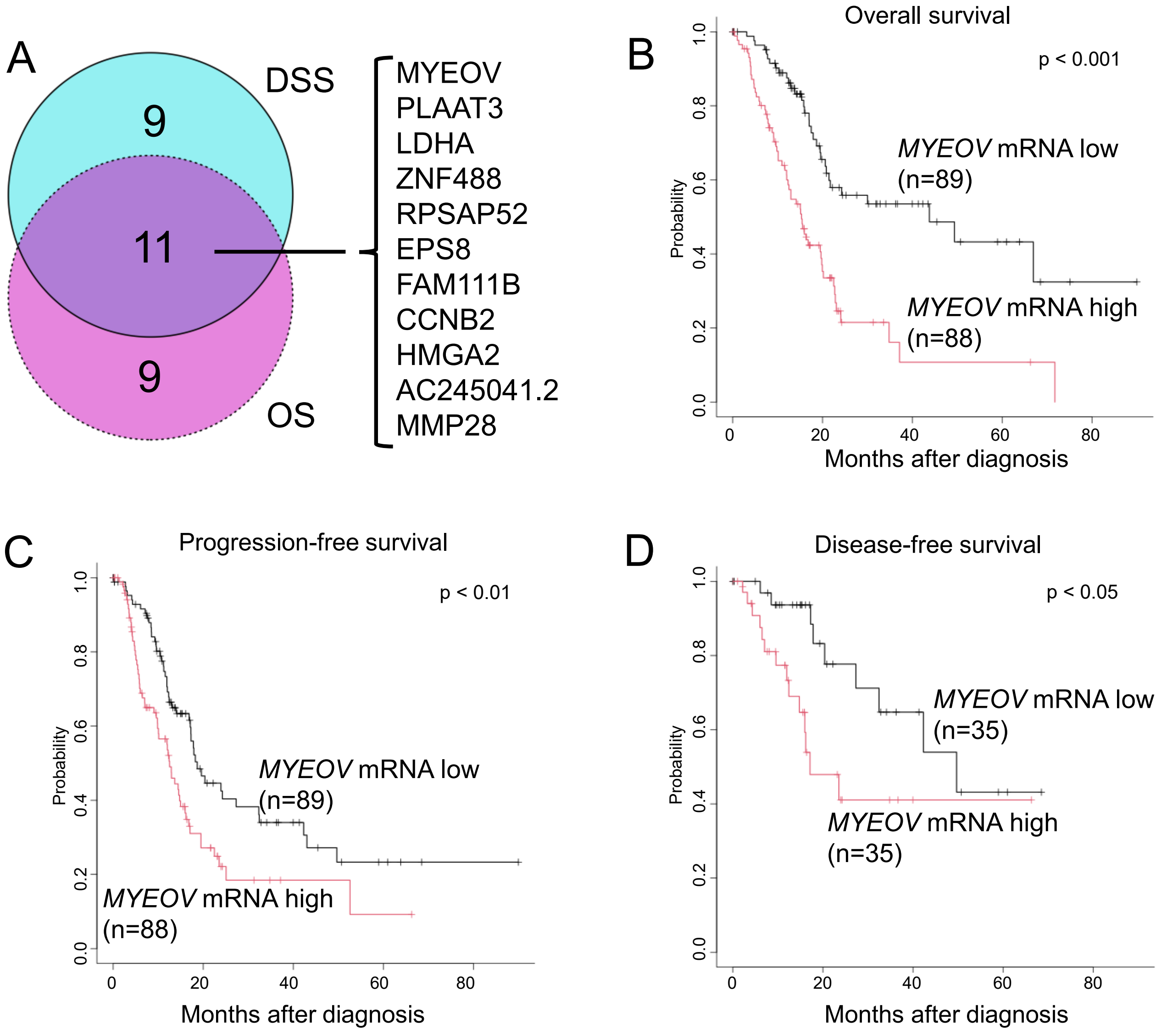

Fig. S1

MYEOV is one of the prognosis indicator genes and is associated with poor clinical outcome. **A**, The top 20 prognostic indicator genes for disease-specific survival (DSS) and overall survival (OS), respectively, were extracted, and presented as Venn diagram. 11 out of 20 genes were overlapped. **B-D**, Kaplan-Meier curve were generated with overall survival (B), progression-free survival (C), and disease-free survival (D), respectively. Patients were divided into two classes using median TPM value of *MYEOV*. P-value was calculated using log-rank test.
