# Supplementary material for: MYEOV overexpression induced by demethylation of its promoter contributes to pancreatic cancer progression via activation of the folate cycle/c-Myc/mTORC1 pathway
Source: BMC Cancer. 2023 Jan 25;23:85. doi: 10.1186/s12885-022-10433-6 (PMC9875418; doi:10.1186/s12885-022-10433-6)
Supplement: Supplementary file 4 — Additional file 4. [file 12885_2022_10433_MOESM4_ESM.pdf]

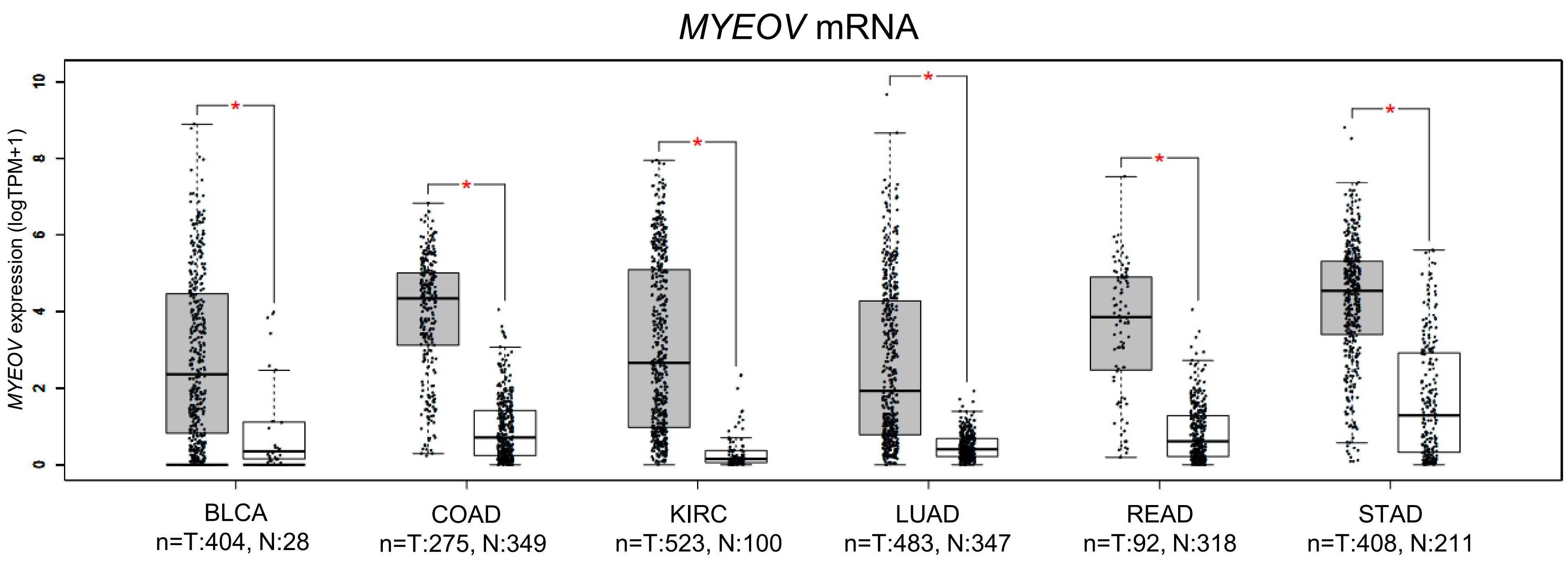

Fig. S2

Pan-cancer expression analyses of MYEOV in 6 cancer types from GEPIA database. Asterisks indicate significant difference ($p<0.01$) of expression between tumor (gray box, T) and non-tumor (white box, N)
