# Supplementary material for: MYEOV overexpression induced by demethylation of its promoter contributes to pancreatic cancer progression via activation of the folate cycle/c-Myc/mTORC1 pathway
Source: BMC Cancer. 2023 Jan 25;23:85. doi: 10.1186/s12885-022-10433-6 (PMC9875418; doi:10.1186/s12885-022-10433-6)
Supplement: Supplementary file 5 — Additional file 5. [file 12885_2022_10433_MOESM5_ESM.pdf]

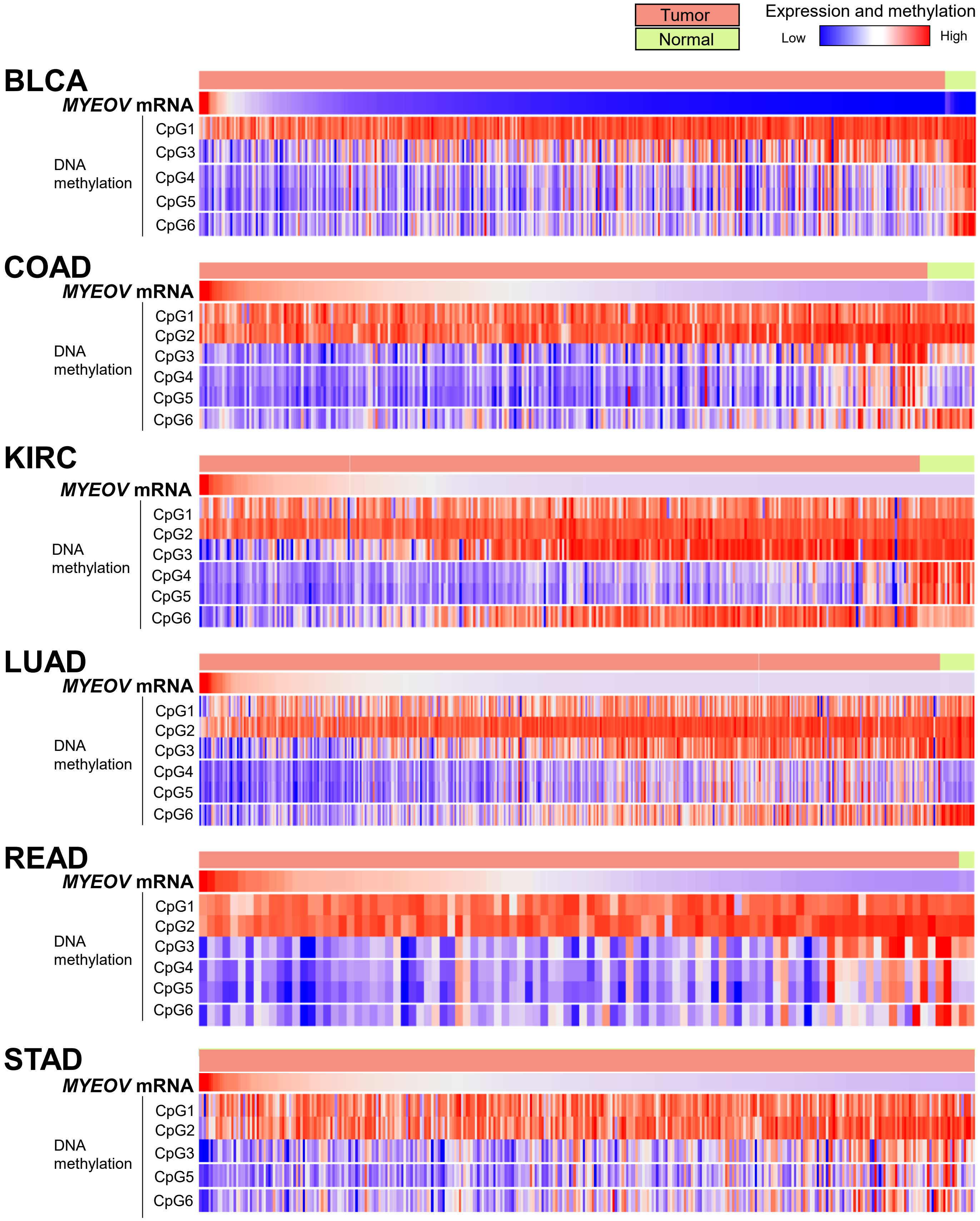

Fig. S3

MYEOV mRNA expression and DNA methylation status in patients of multiple cancer types. Heatmap of pancreatic cancer specimens and non-cancerous tissues of the TCGA data set. The expression of MYEOV and the methylation status of six CpG sites in the region indicated in A are depicted. Each column represents individual specimens. Specimens are sorted in descending order based on TPM values in MYEOV.
