# Supplementary material for: MYEOV overexpression induced by demethylation of its promoter contributes to pancreatic cancer progression via activation of the folate cycle/c-Myc/mTORC1 pathway
Source: BMC Cancer. 2023 Jan 25;23:85. doi: 10.1186/s12885-022-10433-6 (PMC9875418; doi:10.1186/s12885-022-10433-6)
Supplement: Supplementary file 7 — Additional file 7. [file 12885_2022_10433_MOESM7_ESM.pdf]

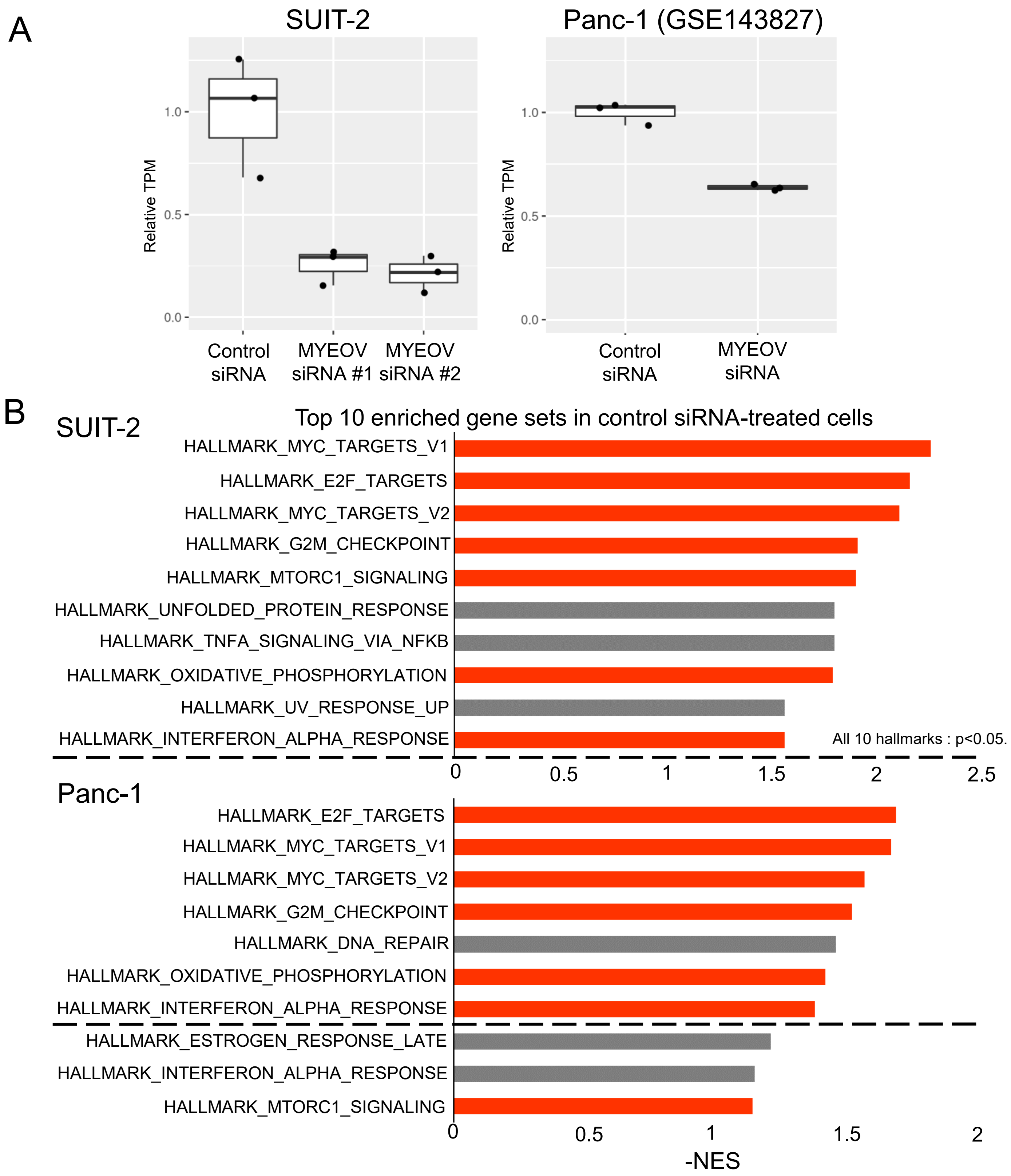

Fig. S4 The comaprison of two RNA-Seq data in *MYEOV*-knockdown pancreatic cancer cells. A, Knockdown efficiency of MYEOV in two RNA-Seq datasets. Relative values based on TPM are presented. B, GSEA analysis was performed on the two indicated datasets. The bar graph shows top 10 enriched gene sets in control siRNA-treated SUIT-2 (upper panel) and Panc-1 (lower panel) cells. Gene sets enriched in both datasets were presented as red bars. In lower panel, hallmarks above the horizontal dashed line indicate statistically significant enrichment (nominal p value < 0.05) in Panc-1 cells.
