# Supplementary material for: MYEOV overexpression induced by demethylation of its promoter contributes to pancreatic cancer progression via activation of the folate cycle/c-Myc/mTORC1 pathway
Source: BMC Cancer. 2023 Jan 25;23:85. doi: 10.1186/s12885-022-10433-6 (PMC9875418; doi:10.1186/s12885-022-10433-6)
Supplement: Supplementary file 11 — Additional file 11. [file 12885_2022_10433_MOESM11_ESM.pdf]

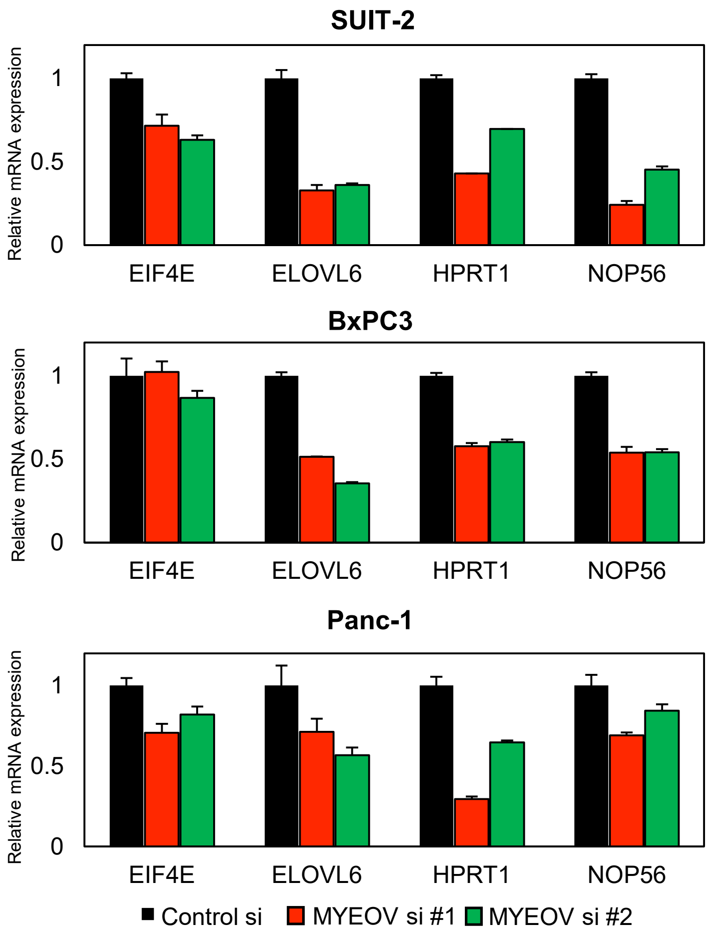

Fig. S5. The expression status of c-Myc target genes upon *MYEOV* knockdown. The expression levels of EIF4E, ELOVL6, HPRT1, and NOP56 were measured by qPCR in the indicated cell lines. Black bars indicate cells transfected with control siRNA, and red and green bars represent cells transfected with MYEOV-targeting siRNA; TBP was used to normalize the expression levels.
