# Supplementary material for: MYEOV overexpression induced by demethylation of its promoter contributes to pancreatic cancer progression via activation of the folate cycle/c-Myc/mTORC1 pathway
Source: BMC Cancer. 2023 Jan 25;23:85. doi: 10.1186/s12885-022-10433-6 (PMC9875418; doi:10.1186/s12885-022-10433-6)
Supplement: Supplementary file 12 — Additional file 12. [file 12885_2022_10433_MOESM12_ESM.pdf]

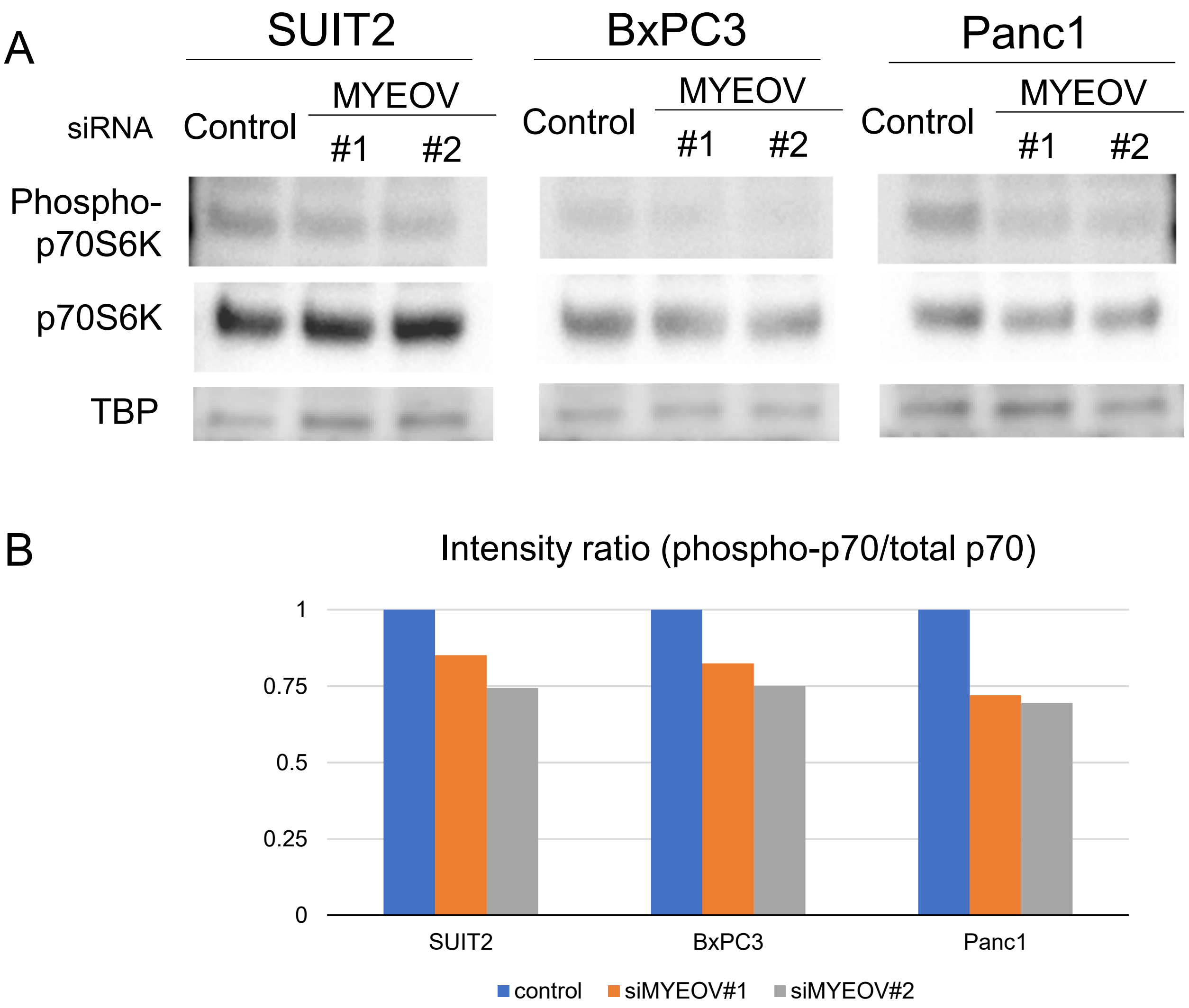

Fig. S6 The effect of MYEOV knockdown on the phosphorylation level of p70S6K, a substrate of mTOR. A, The amounts of total p70S6K protein and their phosphorylation levels were confirmed by Western blot in indicated cell lines. B, Relative phosphorylation intensity of the Western blot signal.
