# Supplementary material for: MYEOV overexpression induced by demethylation of its promoter contributes to pancreatic cancer progression via activation of the folate cycle/c-Myc/mTORC1 pathway
Source: BMC Cancer. 2023 Jan 25;23:85. doi: 10.1186/s12885-022-10433-6 (PMC9875418; doi:10.1186/s12885-022-10433-6)
Supplement: Supplementary file 13 — Additional file 13. [file 12885_2022_10433_MOESM13_ESM.pdf]

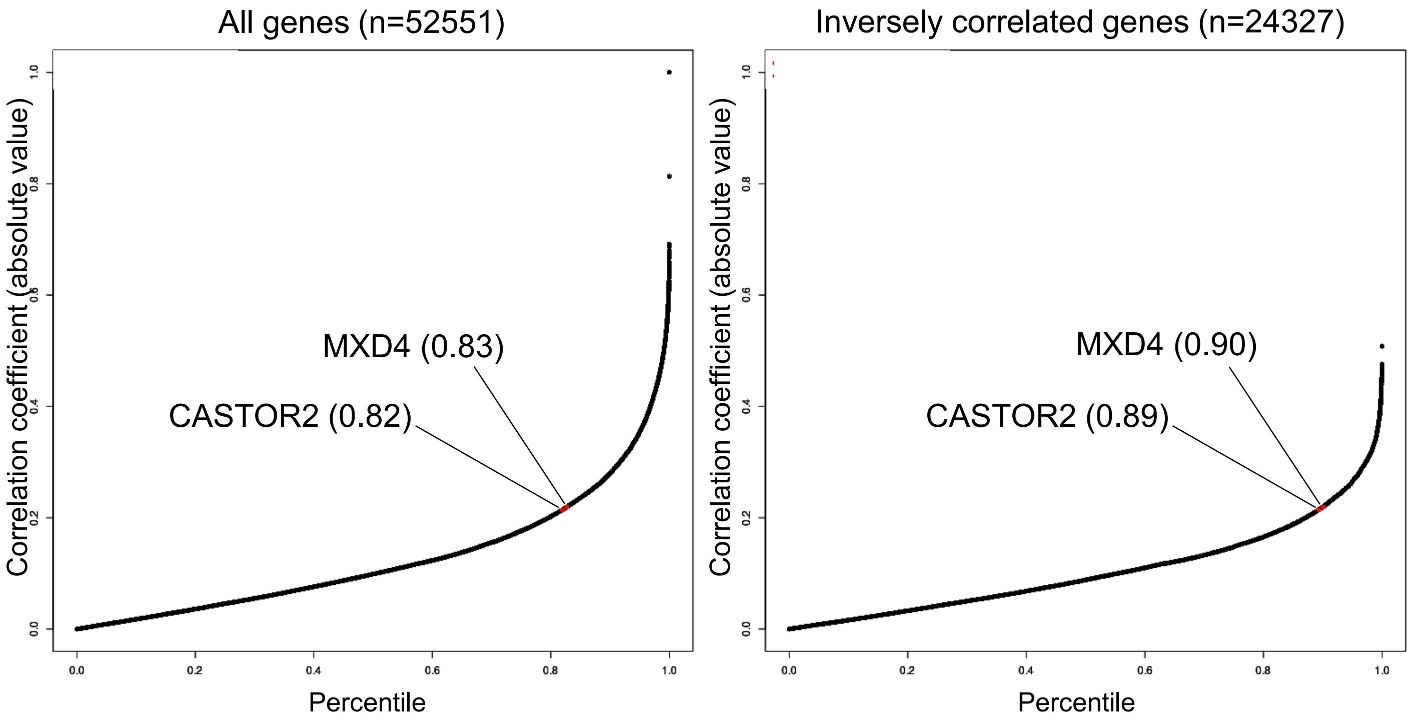


**Fig. S7.** Correlation between MYEOV and MXD4/CASTOR2 expression. In the dataset of pancreatic cancers in TCGA (TCGA-PAAD), Spearman's rank correlation coefficient was calculated between each gene and MYEOV expression using all genes (left panel) or only inversely correlated genes (right panel), and ordered in ascending order. The y axis indicates the absolute values of the correlation coefficients. MXD4 and CASTOR2 are plotted by red dots. Numbers in parentheses indicate the percentile of each gene in ascending order.
