# Supplementary material for: MYEOV overexpression induced by demethylation of its promoter contributes to pancreatic cancer progression via activation of the folate cycle/c-Myc/mTORC1 pathway
Source: BMC Cancer. 2023 Jan 25;23:85. doi: 10.1186/s12885-022-10433-6 (PMC9875418; doi:10.1186/s12885-022-10433-6)
Supplement: Supplementary file 14 — Additional file 14. [file 12885_2022_10433_MOESM14_ESM.pdf]

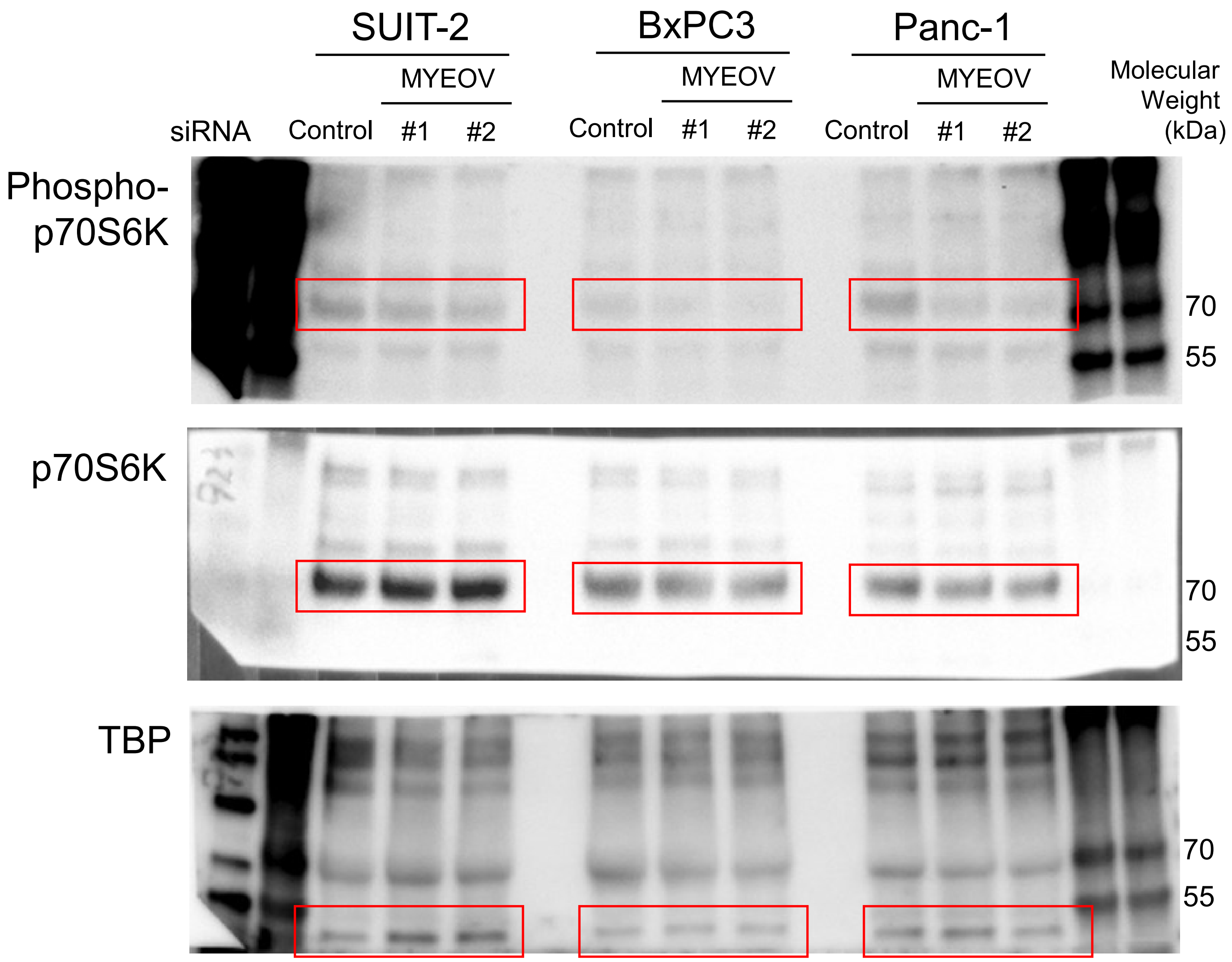

Fig. S8 Original blotting images in Fig. S6. After transfer, the membrane was cut around 50 kDa, and the top half of the membrane was used for the following antibody treatment. Blotting was performed using the same transfer membrane in the following order. First phospho-p70S6K antibody, then loading control antibody, TBP, and finally total p70S6K antibody. After each blotting, the membrane was immersed in stripping solution to remove the antibodies before the next blotting. Bands used in Fig. S6 are indicated by red boxes.
