# Supplementary material for: MYEOV overexpression induced by demethylation of its promoter contributes to pancreatic cancer progression via activation of the folate cycle/c-Myc/mTORC1 pathway
Source: BMC Cancer. 2023 Jan 25;23:85. doi: 10.1186/s12885-022-10433-6 (PMC9875418; doi:10.1186/s12885-022-10433-6)
Supplement: Supplementary file 15 — Additional file 15. [file 12885_2022_10433_MOESM15_ESM.pdf]

Additional file 15 : supplementary figure legends

Fig. S1. *MYEOV* is one of the prognosis indicator genes and is associated with poor clinical outcome. A, The top 20 prognostic indicator genes for disease specific survival (DSS) and overall survival (OS), respectively, were extracted, and presented as Venn diagram. 11 out of 20 genes were overlapped. B-D, Kaplan-Meier curve were generated with overall survival (B), progression free survival (C), and disease-free survival (D), respectively. Patients were divided into two classes using median TPM value of *MYEOV*. P-value was calculated using log-rank test.

Fig. S2. Pan-cancer expression analyses of *MYEOV* in 6 cancer types from GEPIA database. Asterisks indicate significant difference ($p<0.01$) of expression between tumor (gray box, T) and non-tumor (white box, N).

Fig. S3. *MYEOV* mRNA expression and DNA methylation status in patients of multiple cancer types. Heatmap of pancreatic cancer specimens and non-cancerous tissues of the TCGA data set. The expression of *MYEOV* and the methylation status of six CpG sites in the region indicated in A are depicted. Each column represents individual specimens. Specimens are sorted in descending order based on TPM values in *MYEOV*.

Fig. S4. The comaprison of two RNA Seq data in *MYEOV*-knockdown pancreatic cancer cells. A, Knockdown efficiency of *MYEOV* in two RNA-Seq datasets Relative values based on TPM are presented. B, GSEA analysis was performed on the two indicated datasets. The bar graph shows top 10 enriched gene sets in control siRNA treated SUIT-2 (upper panel) and Panc-1 (lower panel) cells. Gene sets enriched in both datasets were presented as red bars. In lower panel, hallmarks above the horizontal dashed line indicate statistically significant enrichment (nominal p-value < 0.05) in Panc-1 cells.

Fig. S5. The expression status of c-Myc target genes upon MYEOV knockdown. The expression levels of *EIF4E*, *ELOVL6*, *HPRT1* and *NOP56* were measured by qPCR in the indicated cell lines. Black bars indicate cells transfected with control siRNA, and red and green bars represent cells transfected with *MYEOV*-targeting siRNA; TBP was used to normalize the expression levels.

Fig. S6. The effect of *MYEOV* knockdown on the phosphorylation level of p70S6K, a substrate of mTOR. A, The amounts of total p70S6K protein and their phosphorylation levels were confirmed by Western blot in indicated cell lines. B, Relative phosphorylation intensity of the Western blot signal.

Fig. S7. Correlation between *MYEOV* and *MXD4* /*CASTOR2* expression. In the dataset of pancreatic cancers in TCGA (TCGA-PAAD), Spearman's rank correlation coefficient was calculated between each gene and *MYEOV* expression using all genes (left panel) or only inversely correlated genes (right panel), and ordered in ascending order. The y axis indicates the absolute values of the correlation coefficients *MXD4* and *CASTOR2* are plotted by red dots. Numbers in parentheses indicate the percentile of each gene in ascending order.

Fig. S8. Original blotting images in Fig. S6. After transfer, the membrane was cut around 50 kDa, and the top half of the membrane was used for the following antibody treatment. Blotting was performed using the same transfer membrane in the following order. First phospho-p70S6K antibody, then loading control antibody, TBP, and finally total p70S6K antibody. After each blotting, the membrane was immersed in stripping solution to remove the antibodies before the next blotting. Bands used in Fig. S6 are indicated by red boxes.
